# Impaired lymphatic function accelerates cancer growth

**DOI:** 10.18632/oncotarget.9953

**Published:** 2016-06-13

**Authors:** Eli Sihn Samdal Steinskog, Solfrid Johanne Sagstad, Marek Wagner, Tine Veronica Karlsen, Ning Yang, Carl Erik Markhus, Synnøve Yndestad, Helge Wiig, Hans Petter Eikesdal

**Affiliations:** ^1^ Department of Biomedicine, University of Bergen, Bergen, Norway; ^2^ Section of Oncology, Department of Clinical Science, University of Bergen, Bergen, Norway; ^3^ Department of Oncology, Haukeland University Hospital, Bergen, Norway

**Keywords:** cancer, lymphangiogenesis, Chy mice, tumor associated macrophages, IL-6

## Abstract

Increased lymphangiogenesis is a common feature of cancer development and progression, yet the influence of impaired lymphangiogenesis on tumor growth is elusive. C3HBA breast cancer and KHT-1 sarcoma cell lines were implanted orthotopically in Chy mice, harboring a heterozygous inactivating mutation of vascular endothelial growth factor receptor-3, resulting in impaired dermal lymphangiogenesis. Accelerated tumor growth was observed in both cancer models in Chy mice, coinciding with reduced peritumoral lymphangiogenesis. An impaired lymphatic washout was observed from the peritumoral area in Chy mice with C3HBA tumors, and the number of macrophages was significantly reduced. While fewer macrophages were detected, the fraction of CD163^+^ M2 macrophages remained constant, causing a shift towards a higher M2/M1 ratio in Chy mice. No difference in adaptive immune cells was observed between wt and Chy mice. Interestingly, levels of pro- and anti-inflammatory macrophage-associated cytokines were reduced in C3HBA tumors, pointing to an impaired innate immune response. However, IL-6 was profoundly elevated in the C3HBA tumor interstitial fluid, and treatment with the anti-IL-6 receptor antibody tocilizumab inhibited breast cancer growth. Collectively, our data indicate that impaired lymphangiogenesis weakens anti-tumor immunity and favors tumor growth at an early stage of cancer development.

## INTRODUCTION

The lymphatic system is a common and early route of metastasis, where the occurrence of lymph node metastasis is a negative prognostic marker. Increased peritumoral lymphatic vessel density (LVD) correlates with increased frequency of lymph node metastasis and worse prognosis in various types of cancer [1–5], pointing to lymphatic vessels as a potential therapeutic target in the treatment of cancer. Various pharmacological inhibitors of lymphangiogenesis are currently investigated clinically [6–8], and the consequences of targeting tumor lymphangiogenesis therefore needs to be explored both in the early and advanced cancer setting. We addressed this issue by studying the early stages of cancer progression in Chy mice, which harbor a heterozygous inactivating point mutation of the tyrosine kinase domain of VEGFR-3 [9]. Importantly, the same mutation has been found in patients with hereditary lymphedema, and this model is therefore of particular interest in a translational context [10, 11]. Chy mice are characterized by impaired lymphangiogenesis in the dermis [10, 12], and accordingly, malignant tumors were implanted orthotopically in the subcutis to evaluate how cancer progression would be affected in a tumor microenvironment where the VEGFR-3 expression level is decreased.

We postulated that impaired lymphangiogenesis could increase the interstitial fluid pressure (IFP) and thus inhibit tumor growth by affecting the blood supply. Alternatively, impaired lymphangiogenesis could prevent tumor antigens from reaching lymph nodes, causing a reduced immune response and weakened tumor immunity. Interestingly, we found accelerated primary tumor growth of C3HBA breast cancer and KHT-1 sarcomas in Chy mice, which was associated with decreased lymphatic washout and a reduced number of peritumoral macrophages. Apart from IL-6, reduced levels of all macrophage-associated cytokines were observed in C3HBA tumors in Chy mice, indicating an impaired innate immune response and potentially influencing the early stages of breast cancer progression.

## RESULTS

### Accelerated tumor growth and impaired peritumoral lymphangiogenesis in Chy mice

**Figure 1:**
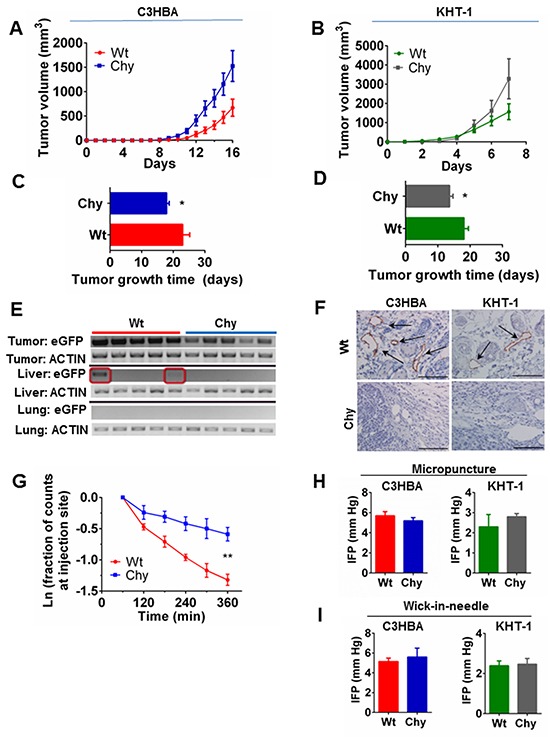
**A–D.** Tumor growth of C3HBA breast cancer and KHT-1 sarcoma in Chy and wt mice. A-B. Tumor growth curves depict the mean tumor volume ± SEM per group, from the day of measureable tumors in the mice. C–D. Days for each tumor to reach 2250 mm^3^ (tumor growth time). Bars display the mean TGT ± SEM per group, demonstrating accelerated tumor growth in Chy mice. C3HBA: n=12 mice/group. KHT-1: n=11 and n=6 in wt and Chy mice respectively.*p<0.05. **E.** Gene expression of the *eGFP*-transgene, which was transduced into C3HBA breast cancer cells implanted in wt and Chy mice. RT-PCR of liver and lung tissue (n=5 mice per group) demonstrates metastatic C3HBA cells actively transcribing *eGFP* in the livers of two out of five wt mice (red boxes). **F.** LYVE-1^+^ lymphatics were present in the peritumoral area of wt mice, but not in Chy mice. Arrows point to LYVE-1^+^ vessels. Scale bars: 100 mm. **G.** Lymphatic washout assessed using Alexa 680-conjugated albumin. The lymphatic washout in the skin overlying C3HBA tumors in Chy mice (n=4) was significantly slower than in wt mice (n=3). **p<0.01. **H.** Intratumoral IFP measured by the micropuncture technique. There was no significant difference in IFP neither in C3HBA nor KHT-1 tumors implanted in wt (n=6 tumors per group) and Chy mice (n=8). Bars depict the mean ± SEM. **I.** Intratumoral IFP measured by the wick-in-needle (WIN) technique. There was no significant difference in IFP neither in C3HBA nor KHT-1 tumors implanted in wt (n=9 and n=7 tumors respectively) and Chy mice (n=2 and n=6 tumors respectively). Bars depict the mean ± SEM.

Accelerated tumor growth of orthotopically implanted C3HBA breast cancer and KHT-1 sarcoma was observed in Chy mice with impaired dermal lymphangiogenesis compared with wt littermates (Figure 1A-1D). Metastases were not seen neither in regional lymph nodes nor in internal organs, when assessed macroscopically and microscopically (data not shown). To detect single-cell metastases, RNA was extracted from lung and liver tissues of mice included in the tumor growth trials, and RT-PCR for *eGFP* positive C3HBA tumor cells demonstrated transgene expression in the liver tissue of two tumor-bearing wt mice, whereas Chy mice had no transgene expression in these organs (Figure 1E). Thus, primary tumor growth was increased but an enhanced metastatic potential was not observed in the Chy model.

Next, we stained the tumor and peritumoral area with a LYVE-1 antibody to assess the lymphatic vessel density in Chy mice. Chy mice had no discernable lymphatics present in the peritumoral area in neither of the two tumor models. Wt mice had on average 30 and 8 LYVE-1 positive lymphatic vessels per hot spot around C3HBA and KHT-1 tumors, respectively (Figure 1F). Apart from a few lymphatic vessels embedded in the outer tumor rim of wt mice, lymphatics could not be identified inside the tumor tissue.

Based on the strong tendency for lymphatic metastasis in the initial stages of breast cancer progression in humans, we assessed whether the missing lymphatics around C3HBA tumors affected lymph flow, measuring washout of labeled albumin by optical imaging [13]. The lymphatic drainage, assessed as washout of Alexa 680-albumin, was significantly lower in the skin overlying C3HBA tumors in Chy mice, compared to wt mice (Figure 1G). The percentage removal of albumin per min from the peritumoral skin of wt mice was: −0.42 ± 0.05 % min^−1^, and Chy mice: −0.18 ± 0.08 % min^−1^ (p=0.005). This demonstrates that lymphatic drainage was strongly impaired in the peritumoral area of C3HBA tumors growing in Chy mice, potentially reducing the drainage of tumor antigens to regional lymph nodes and migration of tumor cells out of the primary tumor bed [14, 15].

We measured tumor IFP by the micropuncture technique [16] in the outer tumor rim to assess whether the impaired peritumoral lymphangiogenesis affected the intratumoral pressure, but there was no significant difference in IFP between wt and Chy mice, neither in C3HBA nor KHT-1 tumors (Figure 1H). Since there may also be a pressure gradient from central to peripheral tumor areas, we measured IFP in the tumor center with the wick-in-needle (WIN) technique [17]. Again we found no significant difference between tumors in wt and Chy mice (Figure 1I). Accordingly, the impaired lymphatic drainage from Chy mice tumors was not caused by changes in intratumoral interstitial fluid pressure.

### Tumor blood vessels and perfusion unaltered by the Chy mutation

**Figure 2:**
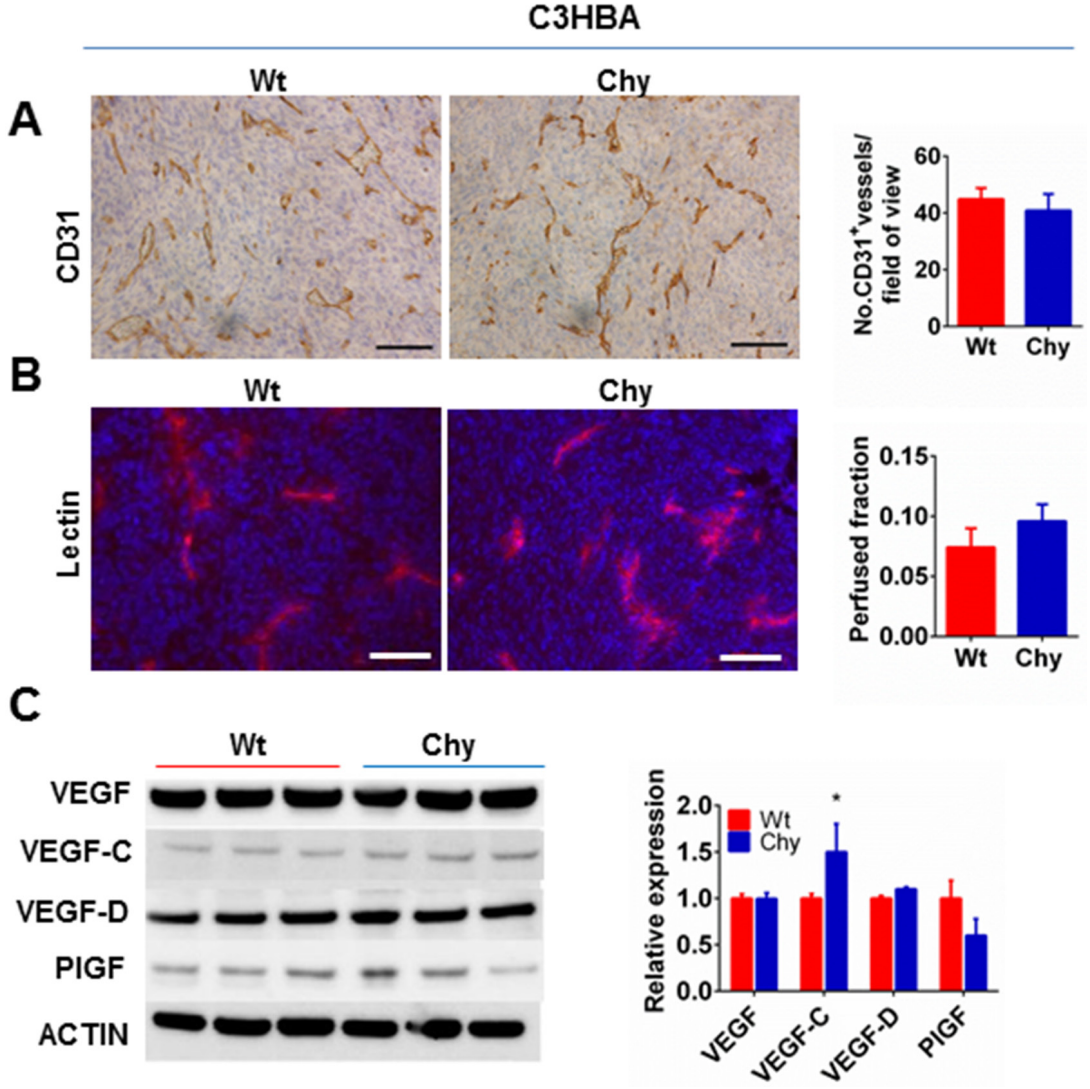
**A.** Immunohistochemistry for CD31 demonstrates no difference in C3HBA intratumoral blood vessel density (BVD) between Chy and wt mice. Scale bars: 100 μm. Bar graph depicts the mean BVD ± SEM, n=3 per group. **B.** Tumor perfusion assessed by i.v. injection of TRITC-conjugated lectin demonstrated no difference between C3HBA tumors in Chy mice and wt mice. Scale bars: 100 μm. Bar graph depicts the mean number of perfused vessels per field-of-view ± SEM, n=7 per group. **C.** Western blot analysis of VEGF family ligands in whole tumor lysate from C3HBA tumors (30 μg protein/lane). VEGF-C is significantly upregulated in Chy compared to wt mice. Densitometry of western blots presented as the ratio of protein of interest over actin expression. Bars depict the mean ± SEM, n=3 per group. *p<0.05.

Based on previous reports, we examined how heterozygous VEGFR-3 inactivation in Chy mice influenced tumor angiogenesis [18]. CD31 staining demonstrated no difference in blood vessel density (BVD) when C3HBA tumors in Chy and wt mice were compared (Figure 2A). To assess the number of perfused blood vessels within Chy and wt C3HBA tumors, lectin was injected through the tail vein and the perfused areas were compared, but no significant difference was found (Figure 2B). Thus, the accelerated primary tumor growth observed in the Chy model was not caused by increased tumor angiogenesis nor increased number of perfused blood vessels.

The influence of VEGFR-3 heterozygous inactivation on ligands of the VEGF family was assessed by protein analysis. VEGF-C was upregulated in C3HBA tumors in Chy mice (Figure 2C), which is likely the result of reduced VEGFR-3 expression in this mouse model. The other VEGFR ligands were not significantly altered (Figure 2C).

### Decreased inflammatory response and macrophage infiltration in Chy mice tumors

**Figure 3:**
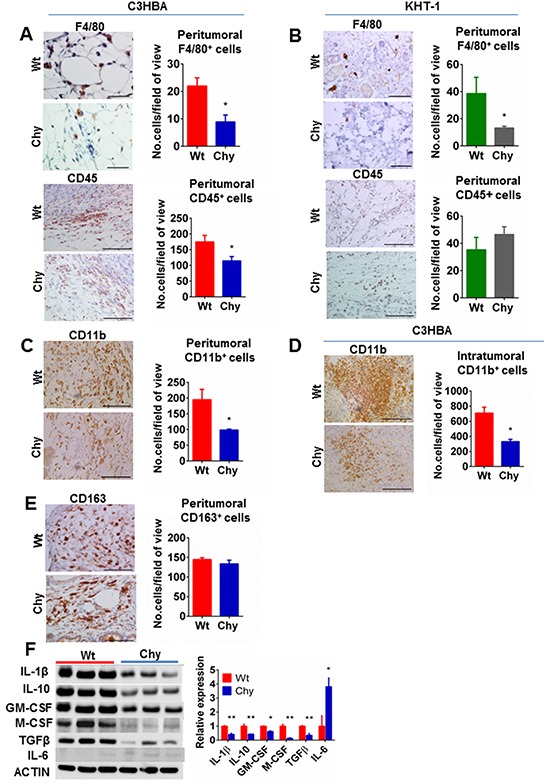
**A–B.** Immunohistochemistry for F4/80 and CD45 in C3HBA breast cancers and KHT-1 sarcomas. (A) Significantly less macrophages (F4/80) and leukocytes (CD45) were observed in the peritumoral area of C3HBA tumors in Chy compared to wt mice. (B) In KHT-1 tumors a significantly reduced number of macrophages, but not leucocytes in general was observed in Chy compared to wt mice. Scale bar: 50 μm. **C–D.** Immunohistochemistry demonstrates significantly fewer peritumoral CD11b^+^ cells surrounding C3HBA (C) and KHT-1 tumors (D) in Chy than in wt mice. Scale bars: 100 μm. **E.** Immunohistochemistry demonstrates no significant difference in peritumoral CD163^+^ cells between Chy and wt mice. Scale bars: 100 μm. Bars graphs depict the mean number of cells per field-of-view ± SEM, n=3 per group. *p<0.05. **F**. Western blot analysis of inflammatory cytokines in whole tumor lysate from C3HBA breast cancers (30 μg protein / lane) demonstrates a global downregulation of pro- and anti-inflammatory cytokines in tumors growing in Chy mice, but an upregulated IL-6 level. Densitometry of western blots presented as the ratio of protein of interest over actin expression. Bars depict the mean ± SEM, n=3 per group. *p<0.05, **p<0.01.

In our assessment of peritumoral lymphatics by LYVE-1 or VEGFR-3 staining (not shown), we observed scattered LYVE-1^+^ or VEGFR-3^+^ cells outside the lymph vessels. Based on the known expression of LYVE-1 or VEGFR-3 by macrophages [19, 20], immunohistochemistry was used to characterize this cell population further. We observed a reduced number of peritumoral F4/80^+^ macrophages in Chy mice and the total number of leukocytes (CD45^+^ cells) (Figure 3A) around C3HBA tumors was significantly reduced in Chy compared to wt mice. For KHT-1 tumors, a reduced number of peritumoral macrophages was observed in Chy mice, whereas the CD45^+^ cell count was no different from wt siblings (Figure 3B). To further assess the innate immune response, staining was performed with a CD11b antibody, which is a well-known marker of myeloid cells, including macrophages. A significant reduction in CD11b^+^ cells in Chy tumors was detected, both within the tumor (Figure 3D) and in the peritumoral area (Figure 3C), in accordance with the F4/80 staining.

The macrophages were further characterized by staining for CD163, a well-known marker of M2 (alternatively activated) macrophages [21]. However, the number of CD163^+^ cells was not significantly different between Chy and wt mice (Figure 3E) and since the total number of macrophages (both M1 and M2) was reduced in Chy mice, this implies that less M1 macrophages were present in the peritumoral tissue of Chy mice, causing a shift towards a higher M2/M1 ratio.

To test the influence of tumor-associated macrophages (TAMs) on early cancer development, wt mice with C3HBA breast cancers were treated with liposomal clodronate before and during tumor initiation to deplete TAMs. Liposomal clodronate treatment inhibited tumor growth, compared to sham treatment (Supplementary Figure S1A), and F4/80 staining demonstrated a reduced number of macrophages (Supplementary Figure S1B). While this points to a pro-tumorigenic role of TAMs in general, liposomal clodronate treatment kills all macrophage subtypes unselectively [22], and do not reflect the Chy phenotype with a skewed M2/M1 ratio. In line with previous data [23], liposomal clodronate treatment reduced tumor angiogenesis (Supplementary Figure S1C), which may have contributed to the impaired tumor growth in this experiment. At the same time, tumor angiogenesis was not affected in Chy mice, demonstrating that liposomal clodronate treatment did not recapitulate the findings in these mice.

Further, we wanted to assess whether the reduced lymphatic washout and increased M2/M1 ratio was associated with altered secretion of macrophage-associated cytokines in Chy mice tumors. A panel of pro- and anti-inflammatory cytokines was examined by protein analysis of whole C3HBA tumor lysates (Figure 3F), and apart from IL-6 all the pro- and anti-inflammatory cytokines were significantly downregulated in Chy tumors. Taken together, our results show a downregulated innate immune response in these mice.

Based on recent data alluding to the important role of tumor infiltrating lymphocytes (TILs) in breast cancers [24], we examined the adaptive immune response by immunostaining for CD3 (T-lymphocytes), CD 20 (B-lymphocytes) and FOX P3 (regulatory T-lymphocytes; Tregs) in C3HBA tumors (Supplementary Figure 2SA-S2C). However, the recruitment of these cell populations was not affected by the Chy mutation, which was also the case for CD3 staining in KHT-1 tumors (Supplementary Figure S2D).

### Increased tumor IL-6 levels in Chy mice and the influence of anti-IL-6 treatment

**Figure 4:**
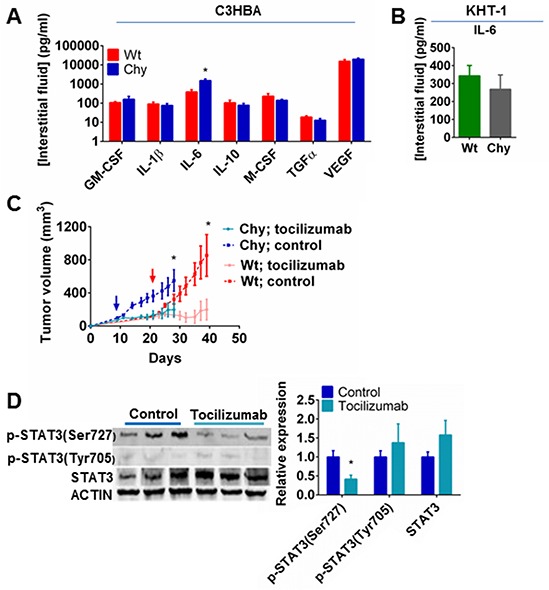
**A.** Multiplex ELISA of tumor interstitial fluid from C3HBA tumors demonstrating significantly higher IL-6 level in Chy compared to wt mice. Bars depict the mean cytokine concentration ± SEM, n=8 per group (except GM-CSF: n=3 per group). *p<0.05. **B.** ELISA of tumor interstitial fluid from KHT-1 tumors demonstrating no significant difference in IL-6 levels between Chy and wt mice. Bars depict the mean cytokine concentration ± SEM, n=5 per group. **C.** Tumor growth of C3HBA breast cancer in Chy and wt mice treated with either IL-6 receptor antibody (tocilizumab) or placebo (saline) i.p for 21 days. The graphs depict the mean tumor volume ± SEM, n=7 per group. Arrows indicate when the treatment started. Tocilizumab significantly reduced tumor growth both in Chy and wt mice. *p<0.05. **D.** Western blot analysis demonstrating reduced STAT3 phosphorylation (Ser727) in C3HBA tumors when IL-6 signaling is inhibited with tocilizumab. Densitometry of western blots presented as the ratio of protein of interest normalized to STAT3 and actin expression. Bars depict the mean ± SEM, n=3 per group. *p<0.05

Since macrophage-associated cytokines were profoundly influenced by the Chy mutation in tumor protein lysates, we evaluated how this affected inflammatory mediators in the tumor interstitial fluid. Multiplex ELISA demonstrated that high concentrations of IL-6 and VEGF were present in the interstitial fluid of C3HBA tumors, with a significantly higher IL-6 level in C3HBA tumors in Chy mice than in wt mice (Figure 4A), thus confirming the western blot analysis for this cytokine. While the western blots measured the total protein content of cytokines within the tumors, the ELISA measured cytokine levels exclusively in the extracellular fluid phase. Thus, the discrepancy between western blots and ELISA for the remaining cytokines assessed may be the result of differential binding of secreted cytokines to extracellular matrix proteins or intracellular sequestration of cytokines that is not detected in the interstitial fluid phase [25, 26]. In KHT-1 sarcomas no difference was detected in IL-6 levels between Chy mice and wt siblings (Figure 4B), which could be due to a high content of necrotic tissue in these tumors, causing spill-over of proteins between the intracellular and extracellular compartments.

Based on the elevated IL-6 levels in C3HBA tumors in Chy mice, both intracellularly and in the tumor interstitial fluid, we assessed the influence of the IL-6 receptor antibody tocilizumab on tumor growth. Treatment with tocilizumab significantly reduced tumor growth both in Chy and wt mice when compared to sham treatment (Figure 4C). Western blot analysis of phosphorylated STAT3 (Ser727) confirmed that tocilizumab blocked IL-6 downstream signaling within the tumors (Figure 4D). This demonstrates that IL-6 is important to C3HBA tumor growth, both in Chy mice and wt siblings, although the profound difference in IL-6 can not alone explain the different growth kinetics.

## DISCUSSION

Increased lymphatic vessel density is associated with enhanced metastatic potential and poor prognosis [1–3]. Interestingly, we found accelerated tumor growth in mice with impaired lymphangiogenesis implanted orthotopically with C3HBA breast cancer or KHT-1 fibrosarcoma. In addition to reduced lymphatic drainage from the C3HBA breast cancers, Chy mice had a reduced number of macrophages, and in particular M1 macrophages recruited to the peritumoral area, indicating a weakened anti-tumor immune response. It is well established that tumor associated macrophages (TAMs) of the M2 subtype contribute to malignant progression and are associated with poor prognosis in patients with *advanced* cancer [27] whereas our results suggest that M1 macrophages could protect against tumor progression in the *early* stages of cancer development as seen in mice without the Chy mutation. Importantly though, these findings should be confirmed in Chy mice with spontaneous cancers in the future.

While the impaired lymphangiogenesis in Chy mice did not affect interstitial fluid pressure inside C3HBA and KHT-1 tumors, lymphatic drainage from the peritumoral area was reduced. This is in line with a previous report in non-tumor bearing Chy mice [28]. Lymphatic peritumoral drainage transports cytokines and antigens to the lymph nodes to elicit an immune response [29], and this process is impaired in mice with reduced lymphangiogenesis [15]. In our study, less macrophages and leukocytes were recruited to the tumor vicinity together with a significant downregulation of macrophage-associated cytokines, pointing to a weakened innate anti-tumor immune response. This is in accordance with recent data demonstrating augmented melanoma and lymphoma growth, as well as decreased local lymph node inflammation, in kCYC mice with reduced lymphangiogenesis [15]. It has been established previously that the humoral immune response on the trunk is not influenced by the Chy mutation [28], and our current finding of no difference in the number of peritumoral adaptive immune cells between Chy and wt mice is in line with these observations.

Macrophages are immune cells characterized by heterogeneity and plasticity. The extremes of macrophage polarization have traditionally been simplified into M1 (classically activated) and M2 (alternatively activated), but the nomenclature in this field is rapidly changing [30]. Evidence from both preclinical and clinical research present a more complex picture of macrophage activation, where macrophage function and balance is skewed depending on the surrounding stimulus [31]. TAMs, which exhibit M2-like features, are known inducers of cancer progression by stimulating angiogenesis, promoting invasion, migration and suppressing antitumor immunity [32, 33]. CD163 is a macrophage-specific scavenger receptor that is upregulated by anti-inflammatory inducers and is therefore associated with macrophages bearing M2 features [34]. We established that the number of CD163 positive macrophages in Chy mice was no different from wt littermates. However, the total number of TAMs was significantly lower in Chy mice, implying a reduced number of anti-tumorigenic M1-activated macrophages to facilitate tumor progression. If tumor macrophage numbers were reduced therapeutically with liposomal clodronate, decreased tumor growth was observed, indicating a pro-tumorigenic effect of macrophages. However, the unselected killing of TAMs with this treatment [22] does not recapitulate the skewed M2/M1 ratio in Chy mice.

In our study, we examined cytokines related to inflammation from two different compartments within the tumor. Whereas the western blot analysis examined the total level of cytokines within the tumor tissue, the assessment of tumor interstitial fluid mirrors the secreted cytokines mediating signals between cells in the extracellular tumor microenvironment. Among seven cytokines analyzed in the C3HBA tumor interstitial fluid, IL-6 was the only cytokine found to be significantly higher in the extracellular microenvironment in Chy tumors. This increase appears to be breast cancer-specific since IL-6 levels were equal in serum and interstitial tissue of non-tumor bearing Chy and wt mice [12], and in KHT-1 tumors with or without the Chy mutation. There are several cell types in the tumor microenvironment that potentially could secrete IL-6; stromal cells (e.g fibroblast, adipocytes) and cancer cells [35, 36]. Indeed, Chy mice are characterized by more subcutaneous adipose tissue than their wt littermates [10]. It has been previously reported that IL-6 is significantly upregulated in peritumoral adipose tissue [37] which could explain a higher IL-6 levels in these mice. The inflammatory microenvironment created by excess adipose tissue has lately received increasing attention, and one of the key cytokines in the link between obesity and cancer is in fact IL-6 [38]. In addition to IL-6 being deposited by the increased adipose tissue, the impaired lymphatic washout in Chy mice could potentially reduce the washout of locally produced cytokines from the tumor microenvironment, thus contributing to the increased IL-6 levels in Chy tumors.

Inflammation is recognized as an enabling characteristic in cancer development [39] and IL-6 is one of several cytokines (IL-1, TNFα, IL-23) found to be essential in inflammatory processes and cancer growth [40, 41]. It has been reported previously that IL-6 induces endothelial cell proliferation [37], but we did not observe increased tumor angiogenesis in Chy tumors. IL-6 further stimulates myeloid-derived suppressor cells (MDSC) [42] and anti-tumor T-cell activity [43]. Furthermore, IL-6 mediates monocytes-to-macrophage differentiation [44, 45], while activating several known macrophage attractants (e.g CCL2, CXCL-12) [46], improving macrophage survival after recruitment [47] and polarizing them into M2 macrophages [47–49]. In the current report, inhibition of IL-6 signaling with tocilizumab yielded significant growth retardation, in orthotopic breast cancer, implicating IL-6 as a key mediator of tumor progression. While tocilizumab treatment inhibited C3HBA tumor growth both in Chy and wt mice, the substantially higher IL-6 level in Chy tumors indicates that this cytokine is relevant for the accelerated tumor growth observed.

Various therapeutic strategies aimed at tumor lymphangiogenesis are currently being tested both preclinically and clinically [6, 8]. In this regard, a pharmacological downregulation of VEGFR-3 would simulate the Chy phenotype, and should be tested in the early cancer setting experimentally. Our results indicate that reduced VEGFR-3 levels could have a detrimental effect on the immunological anti-tumor response in an early stage of primary tumor growth, through impaired lymphangiogenesis and IL-6 elevation. Interestingly, the IL-6 receptor antibody tocilizumab could be employed in this setting to counteract the progression of breast carcinomas.

## MATERIALS AND METHODS

### Mice

Breeding, maintenance and genotyping of heterozygous VEGFR-3 mutant mice (Chy mice) were performed as described previously [10]. The mice were maintained on a C3H background and the breeding yielded either Chy mice (VEGFR3^+/^Chy) or wildtype (wt) siblings (VEGFR-3^+/+^). The mice were anesthetized with a mixture of ketamine (12.2 mg/ml; Ketalar, Pfizer) and metetomidine (24.3 μg/ml; Domitor, Orion Pharma) during micropuncture and wick-in-needle experiments, and with 1 % isofluorane in a combination with O_2_ and N_2_ during all other procedures. Animals were euthanized with CO_2_. All animal experiments were conducted in accordance with the regulations of the Norwegian State Commission for Laboratory Animals, which are consistent with the European Convention for the Protection of Vertebrate Animals used for Experimental and Other Scientific Purposes and Council of Europe (ETS 123). Experiments were performed with the approval from the Association for Assessment and Accreditation of Laboratory Animal Care (AAALAC) International accredited Animal Care and Use Program at University of Bergen.

### Cell lines and tumor growth experiments

The C3HBA breast cancer cell line was purchased from the NCI-Frederick Cancer DCT Tumor Repository (Frederick, MD), whereas the KHT-1 sarcoma was a kind gift from Professor Michael Horsman (Aarhus, Denmark). The tumor cells were grown at 37°C in 5% CO_2_ in Dulbecco's Modified Eagle's Medium (DMEM), supplemented with non-essential amino acids, 10% fetal calf serum, 100 U/ml penicillin, 100 mg/ml of streptomycin and 400 uM L-glutamine (all products: Lonza).

For implantation of mouse tumors, a tumor tissue piece of 1 mm^3^ was excised from an euthanized animal and implanted in the mammary fat pad (C3HBA) or s.c in the dorsal neck region (KHT-1). Female mice of fertile age were used for the C3HBA breast cancer experiment, and male and female mice of fertile age were used for the KHT-1 trial. The C3HBA and KHT-1 tumors are both syngeneic to C3H mice. For the metastasis study, liposomal clodronate and IL-6 experiment, 5 × 10^6^ cells were injected into the lower left mammary fat pad.

Tumor growth was measured at regular intervals using vernier calipers. Tumor volumes were calculated using the formula (a^2^b *(π/6)) where a and b are the shorter and longer diameters of the tumors respectively. The animals were euthanized if they showed signs of serious distress or when tumor diameters exceeded 20 mm, in accordance with ethical guidelines for animal experiments in Norway. The tumor growth time (TGT) was defined as the time taken for tumors to reach 2250mm^3^.

At euthanasia, the tumor tissue was removed for further analysis and the lymph nodes and organs were examined for the occurrence of metastases. Tissue samples were divided in three and either 1) snap-frozen in liquid nitrogen and stored at −80°C, 2) snap-frozen in Tissue-Tek OCT-compound (Sakura) or 3) formalin-fixed and paraffin embedded for immunohistochemistry and subsequent analysis.

To ablate tumor macrophages, wt mice were given intraperitoneal injections every 96 hours of either 200 μl liposomal clodronate or empty liposomes (Encapsula, Nano Science) [50]. Treatment commenced the day before implanting the mice with C3HBA cells, and continued for 21 days.

To assess the functional importance of IL-6, Chy and wt mice were given daily intraperitoneal injections for 21 days with either 200 μl saline (0.9% NaCl) (controls) or 200 μl of an anti-IL-6 receptor monoclonal antibody tocilizumab (RoActemra, Roche) (500 μg/ml) [35].

### Lectin perfusion

Blood vessel perfusion was assessed by i.v. injection of TRITC-conjugated lectin, as described elsewhere [51]. Briefly, 100 μl (1 mg/ml) of TRITC-conjugated *Lecopersicon esculentum lectin* (Vector Laboratories) was injected through the tail vein in anesthetized mice. Ten minutes later, the mice were euthanized by cervical dislocation and perfused with 40 ml phosphate-buffered saline (PBS, Sigma). Tumors were harvested and snap-frozen in OCT medium in liquid nitrogen. Tumor perfusion was analyzed microscopically by measuring the area of lectin positive vessels in three vascular hot spots per tumor at 200x magnification.

### eGFP transfection of the C3HBA breast cancer cell line

The pWPI plasmid (Addgene), containing the *green fluorescent protein (eGFP)* gene was produced in One Shot® Stbl3™ Chemically Competent E. coli (Invitrogen), under growth selection of ampicillin (Pentrexyl, Bristol-Myers Squibb), and positive clones were verified by sequencing. HEK293FT cells (Invitrogen) were co-transfected with envelope plasmid pMD2.g, packaging plasmid psPAX2 (Addgene) and the pWPI plasmid, using Lipofectamine 2000 (Invitrogen). The fully competent lentivirus was harvested and used for transduction of C3HBA cells with pWPI. The *in vitro* transduction efficacy was assessed based on eGFP protein expression using the NucleoCounterÒ NC-3000TM (Chemometec), and the cells were sorted using a BD FACSARIA (BD Biosciences) at the Flow Cytometry Core Facility (University of Bergen) to enrich for eGFP^+^ cells.

### RNA extraction and cDNA production

RNA extraction was performed using the mirVana^TM^ kit (Life Technologies). After DNase treatment (DNA-*free*^TM^ kit, Ambion), the RNA concentrations were measured using a NanoDrop spectrophotometer (Thermo Scientific), and cDNA was made from 500 ng RNA per sample, using the qScript cDNA Supermix (Quanta BioSciences). The cDNA was then treated with 1 μl RNAse H (Invitrogen) to remove remaining RNA. RT-PCR was done using AmpliTaq Gold polymerase, and PCR reactions were run with 35 cycles and the appropriate temperature settings. A *β-actin* PCR reaction was undertaken to ensure equal cDNA content in the samples. All PCR products were checked for specificity by Sanger sequencing.

Primers:

eGFP F2: GAGCTGGACGGCGACGTAAAC

eGFP R2: CACGAACTCCAGCAGGACCATG

β-actin_F1m: TGGCATTGTTACCAACTGGG

β-actin_R1m: AGTTTCATGGATGCCACAGG

### Lymphatic drainage

Lymphatic drainage from the skin directly overlying the tumor was assessed by optically monitoring the depot clearance of near-infrared labelled albumin as described previously [13]. Briefly, 0.5 μl Alexa 680-conjugated bovine serum albumin (Invitrogen) was injected intradermally with a graded Hamilton syringe (34 G), and the mice were optically imaged every 60 min for a 6 hour period using an Optix® MX system (GE Healthcare). Mice were anesthetized (1% isofluorane) only during imaging and remained awake in between measurements. Images were analyzed using eXplore Optix Optiview software (GE Healthcare), and the number of counts calculated for each region of interest. For calculations of depot clearance rates (*k)* the natural logarithm of the fractional amount of counts remaining at the injection site was plotted against time. The resultant monoexponential curves were fitted with linear regression, and *k* found as the slope of each curve.

### Tumor interstitial fluid pressure

Tumor interstitial fluid pressure (IFP) was measured in wt and Chy mice using the micropuncture and wick-in-needle (WIN) techniques, as described previously [17]. Briefly, the IFP in superficial areas (0-1 mm depth) was recorded in anesthetized mice using a sharpened micropipette (diameter; 4-7 μm), connected to a servo-controlled counter-pressure system that was inserted through intact skin (18). To assess the IFP in deep intratumoral areas, a 23 G needle with a 2-4 mm side hole and filled with nylon fibers and saline (WIN technique), was inserted into the central part of the tumor and connected to a transducer dome through a saline filled PE-50 catheter [17].

### Primary antibodies

#### Immunohistochemistry/immunofluorescence

Rabbit polyclonal antibodies: LYVE-1 (Abcam), CD11b (Abcam), CD163 (Santa Cruz), CD3 (Abcam), CD20 (Thermo Fisher), FOXP3 (Abcam). Rat monoclonal antibodies: CD31 (Santa Cruz)), CD45 (R&D, BD Pharmingen), F4/80 (Abcam, AbD Serotec).

#### Western blots

Rabbit anti-actin (Sigma), rat anti-IL1-beta/IL-1F2 (R&D), rabbit anti-TGF-beta (Nordic Biosite), rat anti-GM-CSF (Abcam), rat anti-IL10 (Abcam), goat anti-M-CSF (Abcam), goat anti-VEGFD (Abcam), rabbit anti-VEGF (Abcam), rabbit anti-VEGFC (Abcam), rabbit anti-PlGF (Abcam), rabbit anti-IL-6 (Abcam), rabbit anti-phospho (Ser727) STAT3 (Cell Signaling), rabbit anti-phospho, (Tyr705) STAT3 (Cell Signaling), rabbit anti-STAT3 (Cell Signaling). Anti-GM-CSF, IL-10 and IL-1beta were monoclonal antibodies, all other antibodies were polyclonal.

### Immunohistochemistry of paraffin embedded tissue sections

Tissues were fixed in formalin, paraffin embedded and 4 mm sections prepared. Sections were deparaffinized and rehydrated, before antigen retrieval at 98°C for 1 hour in 0.01 M citrate buffer (pH 6.0). After blocking with diluted serum from the secondary antibody host for 30 min, the slides were incubated overnight (+4°C) with the primary antibody. After blocking endogenous peroxidase activity for 20 min with 3% hydrogen peroxide (Sigma), a biotinylated anti-rat or anti-rabbit secondary antibody (Vector Laboratories) was applied for 30 min as appropriate. The antigen-antibody complex reaction was augmented with avidin-biotin-peroxidase (ABC) for 30 min according to the manufacturer's instructions (Vectastain® ABC Kit, Vector), and stained for 2-10 min with diamino-benzidine tetrahydrochloride (DAB, Vector). The sections were then counterstained with haematoxylin (Fisher), dehydrated and mounted with Entellan (Electron Microscopy Services). Parallel sections were run for all the experiments without primary antibody, to assure the specificity of the immunoreactions.

Blood vessel density (BVD) was assessed by immunolabeling for CD31, using the microvessel density (MVD) method [52]. Briefly, the mean BVD in three vascular hot spots per tumor was assessed at 200x magnification.

Intratumoral macrophages were quantified by immunolabeling with a CD11b antibody, which was the only antibody that yielded acceptable staining within the tumor tissue, while peritumoral macrophages were quantified by immunolabeling with CD11b, F4/80 and a CD163 antibody. CD3, CD20 and FOXP3 antibodies were used to quantify adaptive immune cells in C3HBA and KHT-1 tumors, but extensive necrotic areas within KHT-1 tumors yielded too much unspecific staining for these parameters to be quantified. The number of cells per field-of-view was counted in three hot spots at 200x, 400x or 600x objective magnification.

### Western blot

Cells and tissues were homogenized and lysed in a custom made total protein lysis buffer (50 mM Tris HCl, pH 7.5, 150 mM NaCl, 0.1% SDS, 1% deoxycholate, 1% Triton X-100) containing a protease and phosphatase inhibitor cocktail (Roche, Basel, Switzerland). Protein concentrations were measured by a bicinchoninic acid (BCA) assay (Pierce), and 30 mg protein was loaded per lane for all the immunoblots. The protein lysates were fractionated by electrophoresis using NuPAGE Novex 4-12% Bis-Tris Gel (Invitrogen) and transblotted to nitrocellulose membranes using the XCell II Blot Module (Invitrogen). Adequate sample transfer was confirmed by staining the blot with Ponseau S solution (Sigma). Thereafter the membranes were blocked with 5% bovine serum albumine (Sigma) for 60 min, before immunoblotting overnight with the primary antibody. The immobilized antibody was detected using a horseradish peroxidase-conjugated secondary antibody and stained with SuperSignal West Pico Chemiluminescent Substrate (Thermo Scientific). The immunoreaction was visualized using a LAS-3000 imaging system (FujiFilm, Tokyo, Japan). Immunoblots for actin were made for all samples to assure equal protein loading. The western blot protein bands were compared by automated densitometry using Image J software (NIH, USA).

### Sampling and ELISA of tumor interstitial fluid

Interstitial fluid was sampled from tumors in Chy and wt mice, as described previously [53]. Briefly, the tumor tissue was put on a mesh before centrifugation at 424 *g* for 10 min, yielding 10-50 ml of interstitial fluid. The collected fluid samples were stored at −80°C prior to analysis. The interstitial fluid was assessed for a panel of common inflammatory cytokines using a multiplex fluorescent bead immunoassay kit (Millipore) and an IL-6 ELISA kit (R&D systems), according to our previous protocol [12]. Samples were diluted with serum matrix diluent and run according to the manufacturers' instructions. Total surface fluorescence was measured with a flow-based dual laser system (Luminex^100^, Luminex Corporation). Cytokine concentrations were automatically calculated based on standard curve data.

### Statistics

Data were compared using the nonpaired Student's *t*-test unless otherwise specified. p <0.05 was considered statistically significant. In the tocilizumab treatment study, data were analyzed using two-way analysis of variance (ANOVA).

## SUPPLEMENTARY FIGURES


