# Supplementary material for: Impaired lymphatic function accelerates cancer growth
Source: Oncotarget. 2016 Jun 13;7(29):45789–802. doi: 10.18632/oncotarget.9953 (PMC5216761; doi:10.18632/oncotarget.9953)
Supplement: Supplementary file 1 [file oncotarget-07-45789-s001.pdf]

Impaired lymphatic function accelerates cancer growth

SUPPLEMENTARY FIGURES

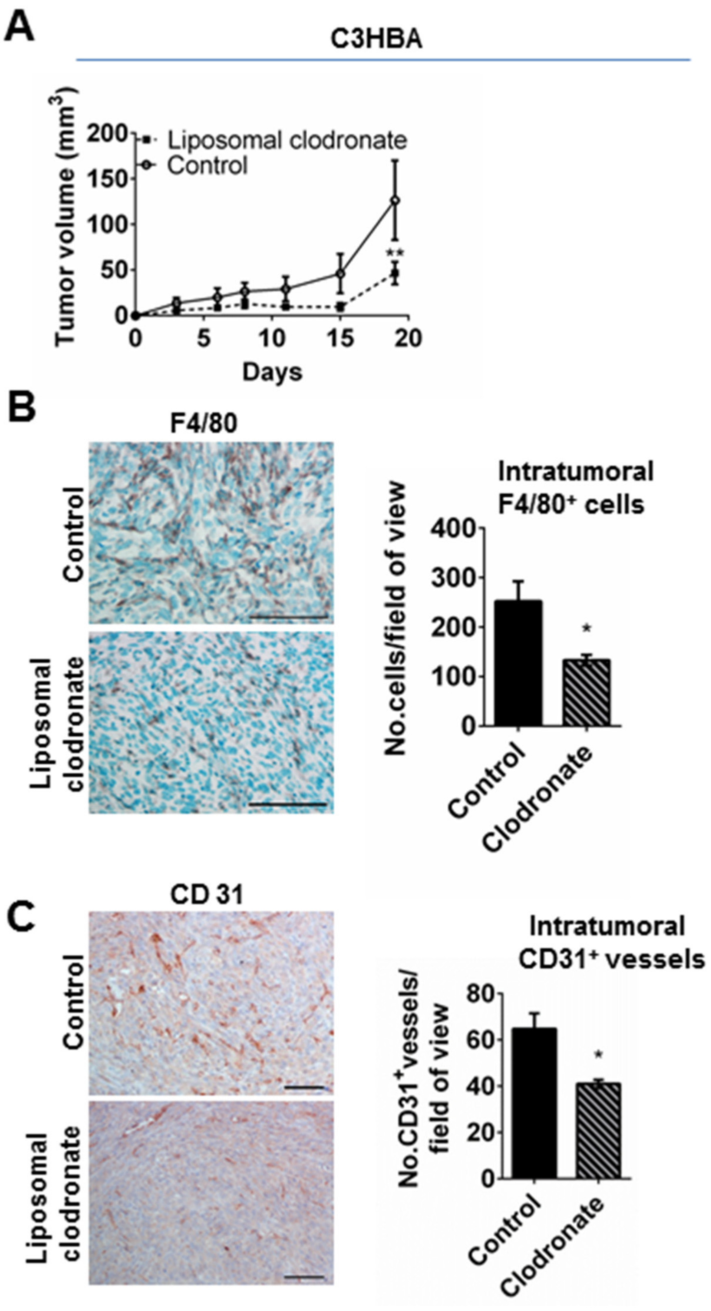

Supplementary Figure S1: A. Growth of C3HBA breast cancers in wt mice, demonstrating significant tumor inhibition during treatment with liposomal clodronate, compared to sham treatment. Treatment commenced the day before C3HBA was injected into the mammary fat pad. Graphs depict the mean tumor volume ± SEM, n=8 per group. **B.** Immunohistochemistry for F4/80 demonstrates significantly less peritumoral macrophages in Chy than in wt mice. Scale bars: 100 µm. Bars graphs depict the mean number of cells per field-of-view ± SEM, n=3 per group. **C.** Immunohistochemistry for CD31 demonstrates significantly less intratumoral blood vessels in Chy than in wt mice. Scale bars: 100 µm. Bar graph depicts the mean blood vessel density (BVD) ± SEM, n=3 per group. *p<0.05. **p<0.01.

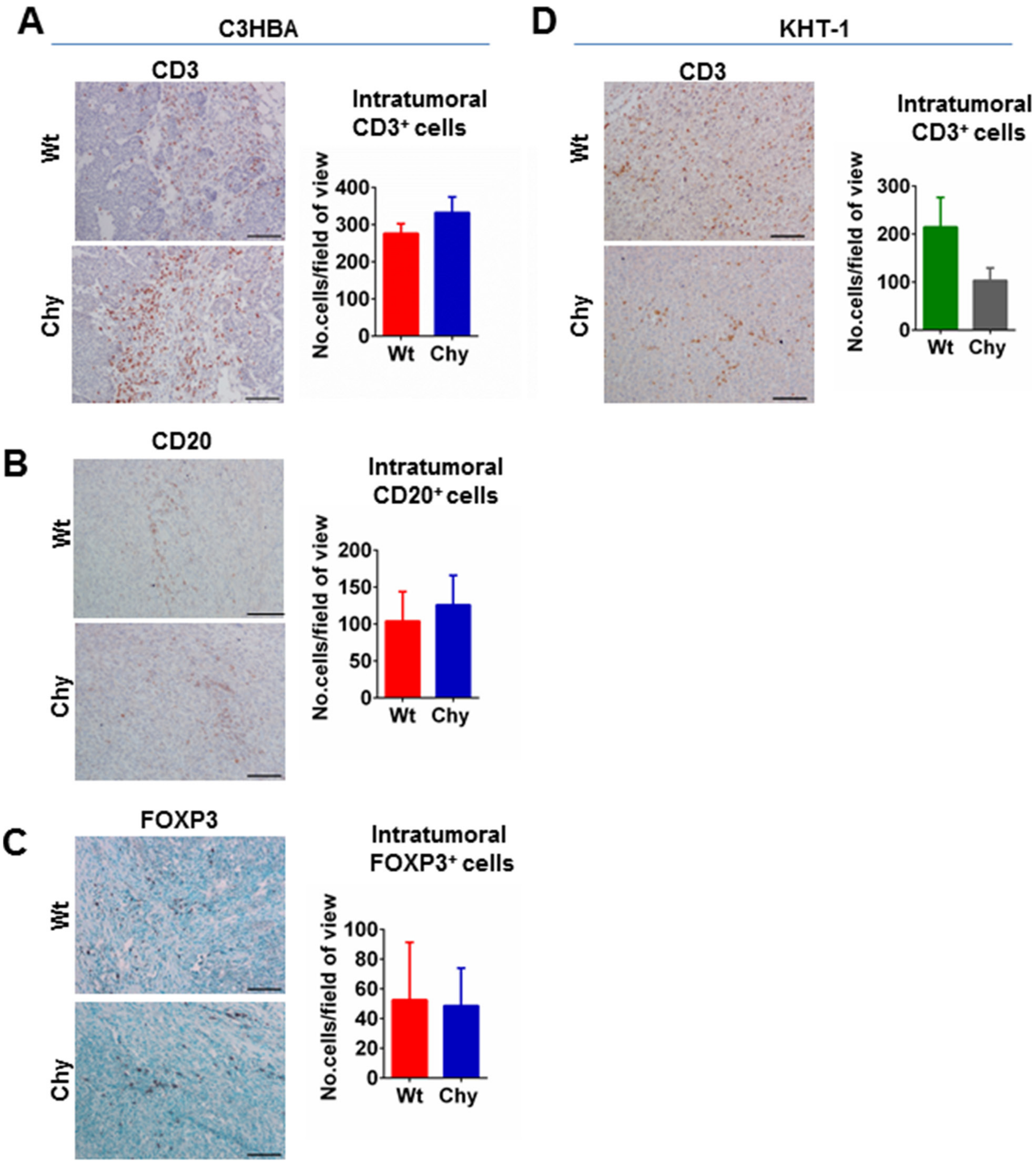

Supplementary Figure S2: A-D. Immunohistochemistry for adaptive immune cells by CD3, CD20 and FOXP3 antibodies. There was no significant difference in the number of intratumoral $CD3^+$, $CD20^+$ or $FOXP3^+$ cells between Chy and wt mice.Bar graphs depict the mean number of cells per field-of-view ± SEM, n=3 per group. Scale bars: 100 μm.
